# Deep learning‐based auto segmentation using generative adversarial network on magnetic resonance images obtained for head and neck cancer patients

**DOI:** 10.1002/acm2.13579

**Published:** 2022-03-09

**Authors:** Daisuke Kawahara, Masato Tsuneda, Shuichi Ozawa, Hiroyuki Okamoto, Mitsuhiro Nakamura, Teiji Nishio, Yasushi Nagata

**Affiliations:** ^1^ Department of Radiation Oncology, Graduate School of Biomedical Health Sciences Hiroshima University Hiroshima Japan; ^2^ Department of Radiation Oncology, MR Linac ART Division, Graduate School of Medicine Chiba University Chiba Japan; ^3^ Hiroshima High‐Precision Radiotherapy Cancer Center Hiroshima Japan; ^4^ Department of Medical Physics National Cancer Center Hospital Tokyo Japan; ^5^ Division of Medical Physics, Department of Information Technology and Medical Engineering, Human Health Sciences, Graduate School of Medicine Kyoto University Kyoto Japan; ^6^ Medical Physics Laboratory, Division of Health Science, Graduate School of Medicine Osaka University Osaka Japan

**Keywords:** CNN, deep learning, GAN, segmentation

## Abstract

**Purpose:**

Adaptive radiotherapy requires auto‐segmentation in patients with head and neck (HN) cancer. In the current study, we propose an auto‐segmentation model using a generative adversarial network (GAN) on magnetic resonance (MR) images of HN cancer for MR‐guided radiotherapy (MRgRT).

**Material and methods:**

In the current study, we used a dataset from the American Association of Physicists in Medicine MRI Auto‐Contouring (RT‐MAC) Grand Challenge 2019. Specifically, eight structures in the MR images of HN region, namely submandibular glands, lymph node level II and level III, and parotid glands, were segmented with the deep learning models using a GAN and a fully convolutional network with a U‐net. These images were compared with the clinically used atlas‐based segmentation.

**Results:**

The mean Dice similarity coefficient (DSC) of the U‐net and GAN models was significantly higher than that of the atlas‐based method for all the structures (*p* < 0.05). Specifically, the maximum Hausdorff distance (HD) was significantly lower than that in the atlas method (*p* < 0.05). Comparing the 2.5D and 3D U‐nets, the 3D U‐net was superior in segmenting the organs at risk (OAR) for HN patients. The DSC was highest for 0.75–0.85, and the HD was lowest within 5.4 mm of the 2.5D GAN model in all the OARs.

**Conclusions:**

In the current study, we investigated the auto‐segmentation of the OAR for HN patients using U‐net and GAN models on MR images. Our proposed model is potentially valuable for improving the efficiency of HN RT treatment planning.

## 1 INTRODUCTION

Head and neck cancer (HNC) is the sixth most common cancer worldwide.^1^ Radiotherapy is offered to 75% of patients.^2^ The treatment technique has been advanced from 3D‐conformal radiotherapy to intensity‐modulated radiation therapy (IMRT).^3^ Specifically, IMRT can permit dose coverage of target volumes by reducing the dose for organs at risk (OARs).^4^ Thus, it is important to accurately delineate the target volume and OAR.^5^ There are many OARs, including the parotid glands, submandibular glands, and optic nerves. An accurate segmentation is required to ensure effective and safe patient treatment. A manual delineation for the segmentation of the target volume and OAR is labor‐intensive and time‐consuming. Based on a previous study, the segmentation for each HNC patient undergoing IMRT requires an average of 2.7 h.^6^

To reduce the stress and time consumption involved in manual segmentation, an auto‐segmentation system has been developed. Atlas‐based auto‐segmentation has already been established by several vendors.^7^ This technique decreases the amount of time required for segmentation by 30%–40% when compared to manual segmentation.^8^ However, atlas‐based auto‐segmentation uses a fixed size. Hence, this limits its ability to adapt to the difference in patient anatomy.^9^

Artificial intelligence (AI)‐based methods have recently been proposed for the segmentation required for treatment planning. AI‐based algorithms can perform highly intensive computations. Thus, auto‐segmentation can be completed within a short time after the training model is created. An AI‐based auto‐segmentation is desirable for replanning and adaptive radiotherapy (ART). Several machine learning‐based algorithms, such as random forest‐based, support vector machine (SVM)‐based, and deep learning (DL)‐based methods, have been used for HN multi‐organ segmentation.^10^, ^11^, ^12^, ^13^ For DL‐based methods, convolutional neural networks (CNNs) have generally been used for segmentation. Ibragimov et al. proposed the first DL‐based algorithm for HN segmentation in OARs using CT images.^14^

Magnetic resonance imaging (MRI) can provide a higher contrast for soft tissue than CT without radiation exposure. An MR‐based radiation treatment planning has been performed with the help of recent advanced developments such as MR‐Linac.^15^, ^16^, ^17^ Recently, ART has been developed to modify the treatment plan for weight loss and target shrinkage during treatment.^18^ MR‐guided RT (MRgRT) can modify radiotherapy plans according to changes in patient anatomy assessed by daily MRI.^19^ To realize MRgRT, rapid delineation of the target and OAR should be performed. In several previous studies, MRI‐based segmentation with CNNs has been proposed for HN patients.^20^, ^21^ In these studies, a 3D CNN was used for the segmentation of tumor regions in the brain and HN.

General adversarial networks (GANs) have proved successful in image synthesis. The GAN uses two networks that enhance each other's performance by performing competitive and iterative training. Dong et al. reported that GAN improved the accuracy of thorax segmentation.^22^ However, GAN has not been used for the segmentation of HN patients.

The current study proposes an auto‐segmentation model using GAN using a patch segmentation. Moreover, we compare the GAN model with the conventional models for HN segmentation.

## 2 MATERIALS AND METHODS

### 2.1 Data

In the current study, 55 sets of HN MRI images were obtained for tissue segmentation from the American Association of Physicists in Medicine annual meeting auto‐segmentation grand‐challenge (RT‐MAC) 2019.^24^ The data were split into a 40/15 training and validation dataset. Specifically, patients who underwent treatment at the University of Texas MD Anderson Cancer Center between 2017 and 2018 were selected. The patients included 50 men (91%) and five women (5%) with a median age of 63 years (range: 32–77 years).

### 2.2 MRI scan

T2‐weighted scans were acquired using a single 1.5 T Siemens MAGNETOM Aera MRI scanner (Siemens Healthcare, Erlangen, Germany). All scans were acquired using a multiple two‐dimensional (2D) turbo spin‐echo sequence. The acquisition parameters corresponded to refocusing pulse = 180${}^{\circ }$, echo time = 80 ms, repetition time = 4800 ms, flip angle = 90${}^{\circ }$, slice thickness = 2.0 mm, pixel bandwidth = 300 Hz, matrix size = 512 × 512, and field of view = 256 × 256 mm^2^.

### 2.3 Manual delineation of target structures

Normal tissue was segmented on T2‐weighted images by a radiation oncologist with over 10 years of clinical experience (ASRM). Each MRI scan covered the entire HN area, and manual segmentations were performed according to the consensus guidelines.^25^ The targets of the segmentations were lymph node levels II and III, parotid glands, and submandibular glands. The details of the segmentations were demonstrated in Kieselmann et al.^26^ The interobserver variability of the segmentation was evaluated by three observers with sufficient clinical experience of medical physicist and dosimetrists.

### 2.4 Fully CNN

**FIGURE 1:**
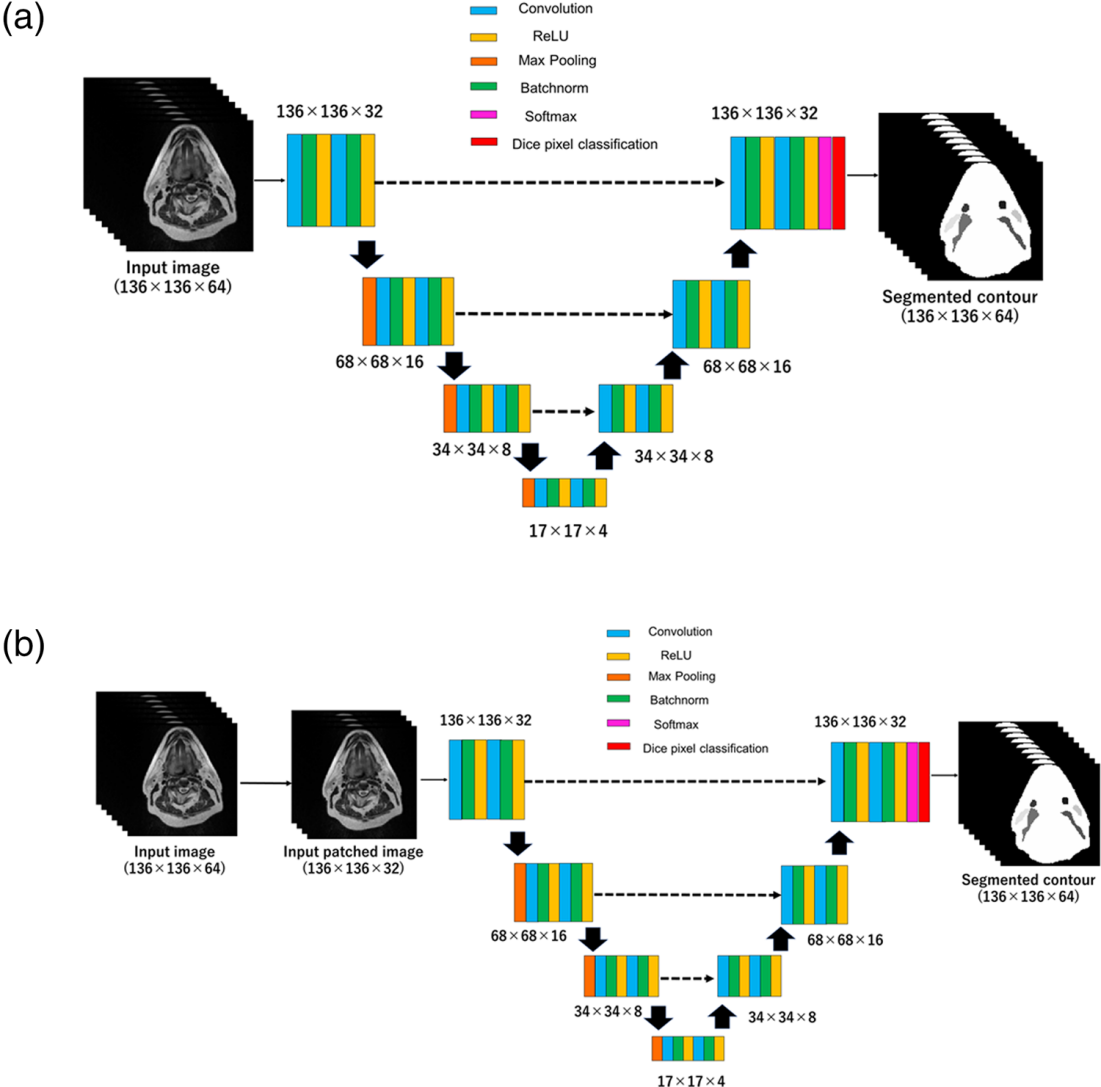
2.5D U‐net (a) and 3D U‐net (b) for head and neck segmentation

Conventionally, a 2D CNN, generally utilized for pattern recognition and image classification, is used for segmentation. It operates with 2D input and 2D filters. Zhang et al. proposed a multimodal network with various MRI images inputted for red‐green‐blue (RGB) channels.^27^ The 3D CNN, which uses 3D input images and 3D filters, fully utilizes the advantages of spatial information and can train using images up to the voxel level. Urban et al. demonstrated the feasibility of the 3D CNN for segmenting brain tumors.^28^ The approaches involving 2.5D were introduced by Moeskops et al.^29^ In these approaches, three orthogonal 2D patches were used in the XY, YZ, and XZ planes. The 2.5D CNN exhibits the advantage of more spatial information with less computational cost when compared to the 3D CNN. In another 2.5D CNN approach, a patch of multiple slices was used for the input image during training. The U‐net uses a fully convolutional neural network (FCN) with a skip connection. In the current study, we used a 3D U‐net that can efficiently segment arbitrarily voxel‐sized images. Moreover, we evaluated the augmentation effect using a 2.5D U‐net that uses a random patch of multiple slices by comparing it with the 3D U‐net. A detailed network of the 3D U‐net and 2.5D U‐net is shown in Figure 1. The size of the MRI image was set to 512 × 512 × 130 mm^3^, which was resized to 136 × 136 × 64 mm^3^ for training. The 3D U‐Net was trained on full‐sized 3D volumes. With respect to the 2.5D U‐net, the patch size was 136  ×  136  ×  32 mm^3^. The patch size was determined as the minimum size that the patched image included in segmentation. The 2.5D U‐Net was trained with multi‐slice image volumes. All the U‐net models comprised a total of 59 layers containing 12 convolution layers, three max‐pooling layers, 19 batch normalization layers, 18 activation layers, and Dice pixel classification. All activation layers were rectified linear units (ReLUs). The kernel sizes were set to 3  ×  3  ×  3 for all the convolution layers. Furthermore, upsampling of the low‐resolution images was performed using a transposed convolution layer with kernel sizes of 2  × 2  ×  1 and 2  × 2  ×  2. The ReLU removes output values below 0 at the output features and makes learning with images more efficient. The loss function was employed using the Dice loss function.

### 2.5 GAN

**FIGURE 2:**
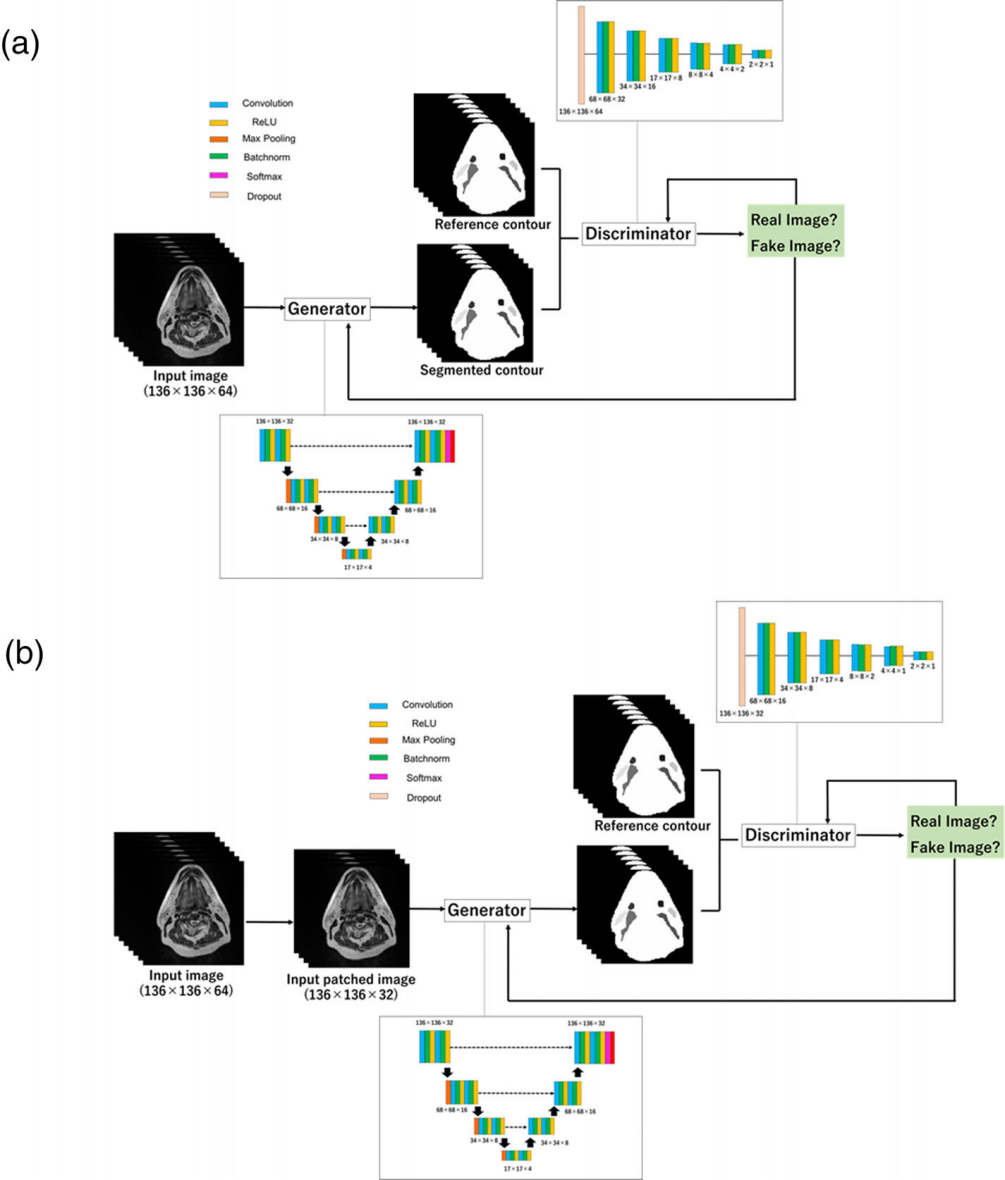
2.5D generative adversarial network (GAN) architecture (a) and 3D GAN architecture (b) for head and neck segmentation

In the current study, we implemented an auto‐segmentation model using a 3D GAN and 2.5D GAN. The 2.5D GAN used a random patch of multiple slices, which was similar to that used by the 2.5D U‐net. An overview of the 3D and 2.5D GAN models is shown in Figure 2.

The GAN includes a generator to estimate the segmentation and a discriminator to distinguish the reference segmentation from the generated segmentation. The generator attempts to produce realistic segmentations that confuse the discriminator. The generator of the 3D GAN model for the 3D U‐net and 2.5D model GAN uses the 2.5D U‐net. The discriminator uses the FCN, which has six convolution layers for extracting features from image and product the output image. These two networks were simultaneously trained. With respect to the training dataset, the hyperparameters were optimized. Specifically, it was adjusted to one for each algorithm of the test dataset. The generator loss was computed as the sum of the weighted cross‐entropy loss of the contour images and mean squared error of the residual images. The weighted cross‐entropy was used as the discriminator loss. To minimize these losses, an Adam optimizer was applied. The 3D GAN was trained with 200 epochs, and the 2.5D GAN was trained with 80 epochs and 30 patches. The proposed models were implemented using MATLAB (v. 2019b, MathWorks, Inc., MI, USA) on a 12‐GB NVIDIA GeForce RTX 3090 Graphics Processing Unit (GPU).

### 2.6 Atlas‐based segmentation

The atlas‐based segmentation used commercial atlas‐based segmentation software Velocity AI (Velocity Medical Systems, Atlanta, Georgia). An automatic segmentation with single atlases of the HN cancer data was performed using T2‐weighted MRI images.

### 2.7 Evaluation metrics

The accuracy of the auto‐segmentation was evaluated by comparing it to the manual segmentation, which corresponds to the gold standard for the validation dataset.^30^, ^31^ The degree of coincidence of the manual segmentation and auto segmentation with atlas or DL methods was assessed using mean Dice similarity coefficient (DSC), mean Jaccard similarity coefficient (JSC), and maximum Hausdorff distance (HD) (unit: mm).

The DSC measures the volumetric overlap between the manual and auto‐segmentation,^31^, ^32^ which is calculated as follows:

$$\mathrm{DSC}=\frac{2\left|A\cap B\right|}{\left|A\right|+\left|B\right|}$$

where A is used manual segmentations, and B is the segmentations obtained with auto‐segmentations. The DSC produces output values between 0 and 1, where 1 denotes two perfectly coincidental contours, and 0 denotes two contours with no coincidence. The JSC calculates the ratio of the intersection volume and entire union volume of the manual segmentation and auto‐segmentation^32^; it is calculated as follows:

$$\mathrm{JSC}=\frac{\left|A\cap B\right|}{\left|A\cup B\right|}$$

where A is used manual segmentations, and B is the segmentations obtained with auto‐segmentations. The JSC is also situated between 0 and 1, wherein 1 indicates perfect coincidence, and 0 indicates no coincidence. The maximum HD measures the maximum distance of a point in a set manual segmentation to the nearest point in a second set of auto‐segmentation.^33^

$$\mathrm{HD}=\max\left(h\left(A,B\right),h\left(B,A\right)\right)$$

$$h\;\left(A,B\right)=\max_{a\in A}\min_{b\in B}\left\|a-b\right\|$$

where A is used manual segmentations, and B is the segmentations obtained with auto‐segmentations. Specifically, ||a–b|| denotes the Euclidean distance between a and b, which are points on the boundary of manual segmentation and auto‐segmentation. Furthermore, h (A, B) is termed as directed HD. A smaller HD suggests higher coincidence of the segmentations.

To evaluate the segmentation accuracy, a *t*‐test was performed to compare the differences between the reference segmentation and atlas‐based or DL‐based methods. The level of significance was set at *p* < 0.05 in statistical analyses.

## 3 RESULTS

**FIGURE 3:**
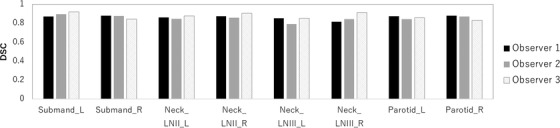
The mean Dice similarity coefficient (DSC) values between the manual segmentation data included in RT‐MAC 2019 dataset and manual segmentation by three radiation observers

Figure 3 showed the DSC measured the volumetric overlap between the manual segmentation data included in RT‐MAC 2019 dataset and manual segmentation by 3 radiation observers. The average DSCs were 0.87 for the left submandibular gland, 0.88 for the right submandibular gland, 0.86 for the left lymph node levels II, 0.87 for the right lymph node levels II, 0.85 for the left lymph node levels III, 0.81 for the right lymph node levels III, 0.88 for the left parotid gland, and 0.89 for right parotid gland.

**FIGURE 4:**
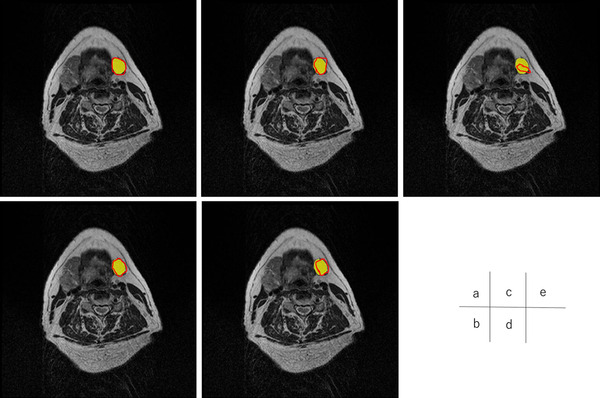
Comparison of manual segmentation and (a) 2.5D generative adversarial network (GAN), (b) 3D GAN, (c) 2.5D U‐net, (d) 3D U‐net, and (e) atlas‐based method in the left submandibular segmentation. The yellow region denotes the reference segmentation, and red line denotes the segmentation by atlas‐based or deep learning methods

**FIGURE 5:**
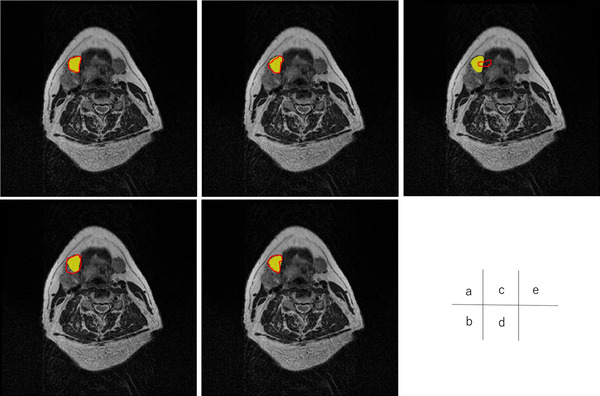
Comparison of manual segmentation and (a) 2.5D generative adversarial network (GAN), (b) 3D GAN, (c) 2.5D U‐net, (d) 3D U‐net, and (e) atlas‐based method in the right submandibular segmentation. The yellow region denotes the reference segmentation, and red line denotes the segmentation by atlas‐based or deep learning methods

**FIGURE 6:**
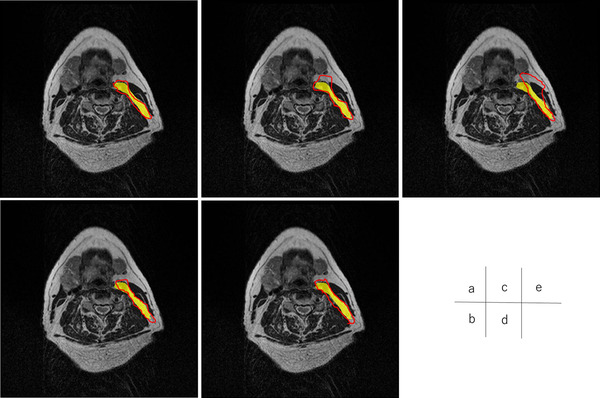
Comparison of manual segmentation and (a) 2.5D generative adversarial network (GAN), (b) 3D GAN, (c) 2.5D U‐net, (d) 3D U‐net, and (e) atlas‐based method in the left lymph node levels II. The yellow region shows the reference segmentation, and red line shows the segmentation by atlas‐based or deep learning methods

**FIGURE 7:**
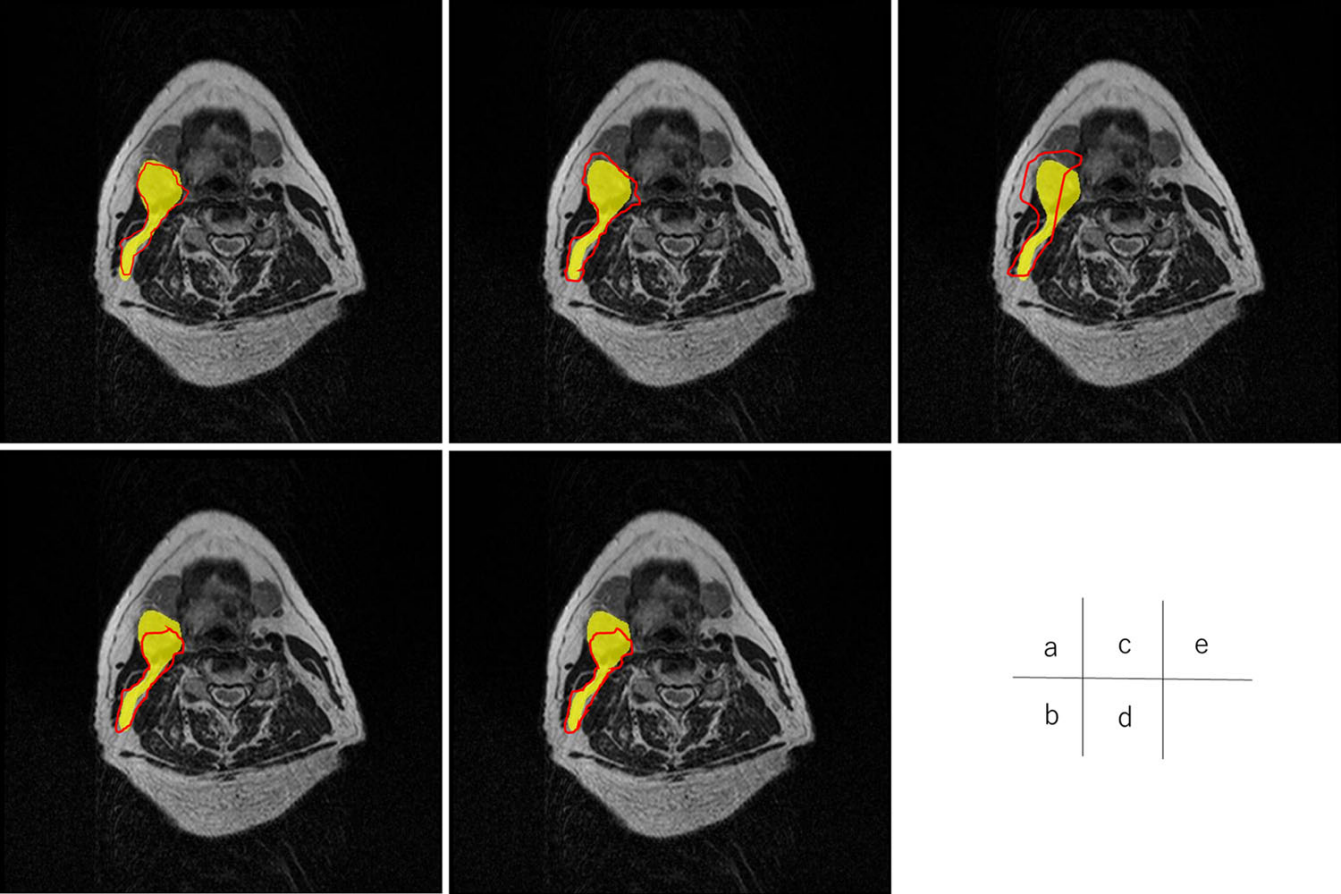
Comparison of manual segmentation and (a) 2.5D generative adversarial network (GAN), (b) 3D GAN, (c) 2.5D U‐net, (d) 3D U‐net, and (e) atlas‐based method in the right lymph node levels II. The yellow region denotes the reference segmentation, and red line denotes the segmentation by atlas‐based or deep learning methods

**FIGURE 8:**
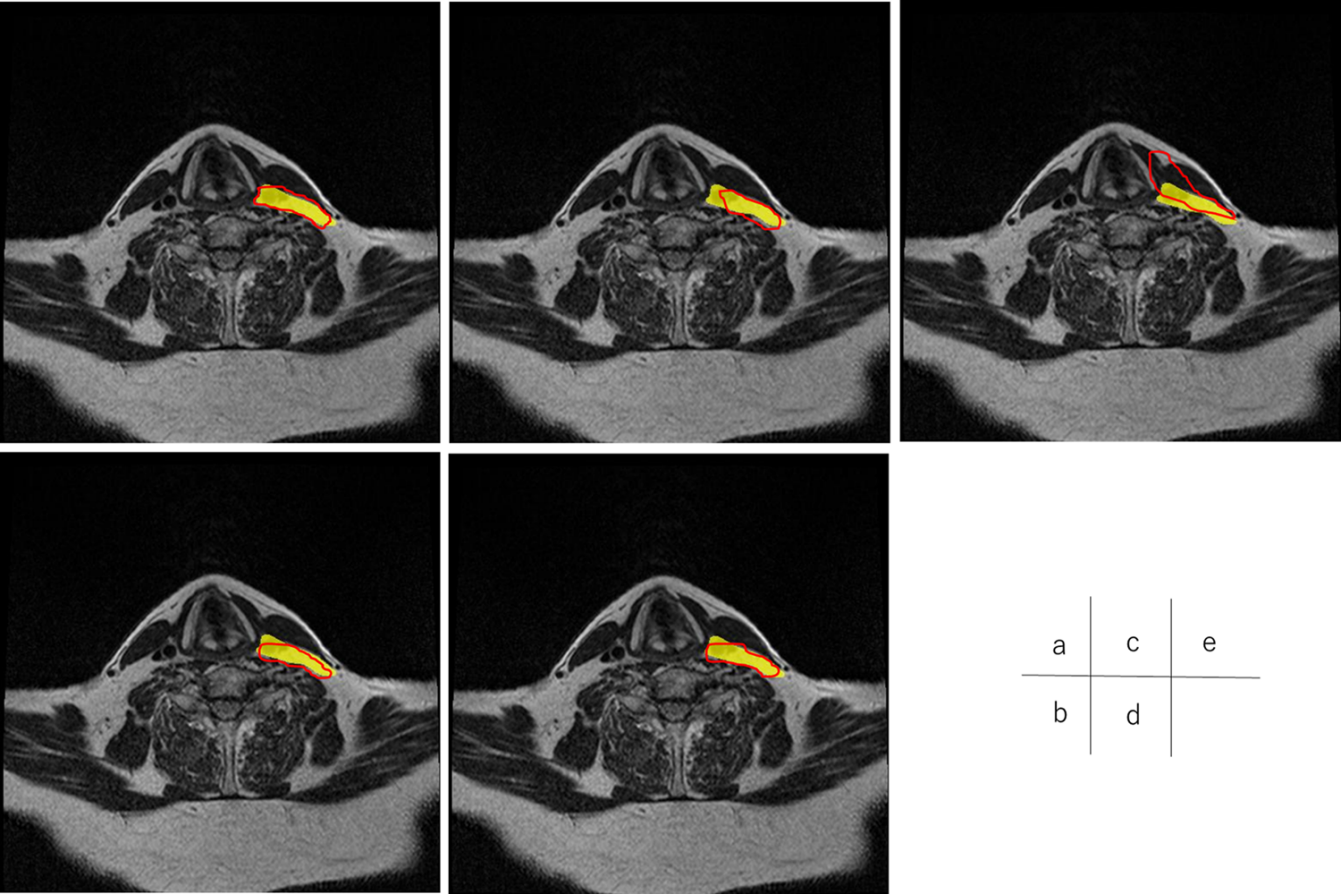
Comparison of manual segmentation and (a) 2.5D generative adversarial network (GAN), (b) 3D GAN, (c) 2.5D U‐net, (d) 3D U‐net, and (e) atlas‐based method in the left lymph node levels III. The yellow region denotes the reference segmentation, and red line denotes the segmentation by atlas‐based or deep learning methods

**FIGURE 9:**
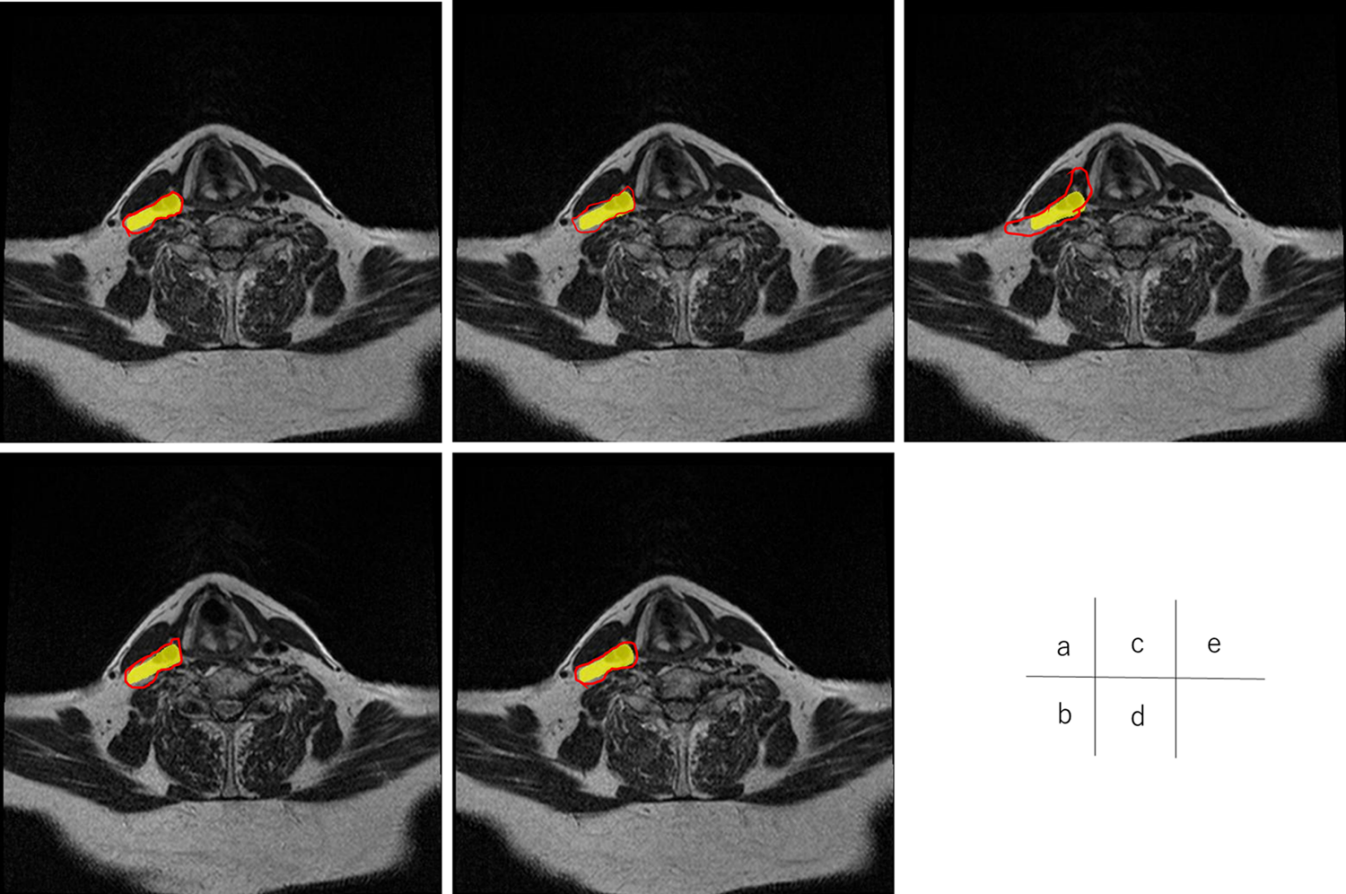
Comparison of manual segmentation and (a) 2.5D generative adversarial network (GAN), (b) 3D GAN, (c) 2.5D U‐net, (d) 3D U‐net, and (e) atlas‐based method in the right lymph node levels III. The yellow region denotes the reference segmentation, and red line denotes the segmentation by atlas‐based or deep learning methods

**FIGURE 10:**
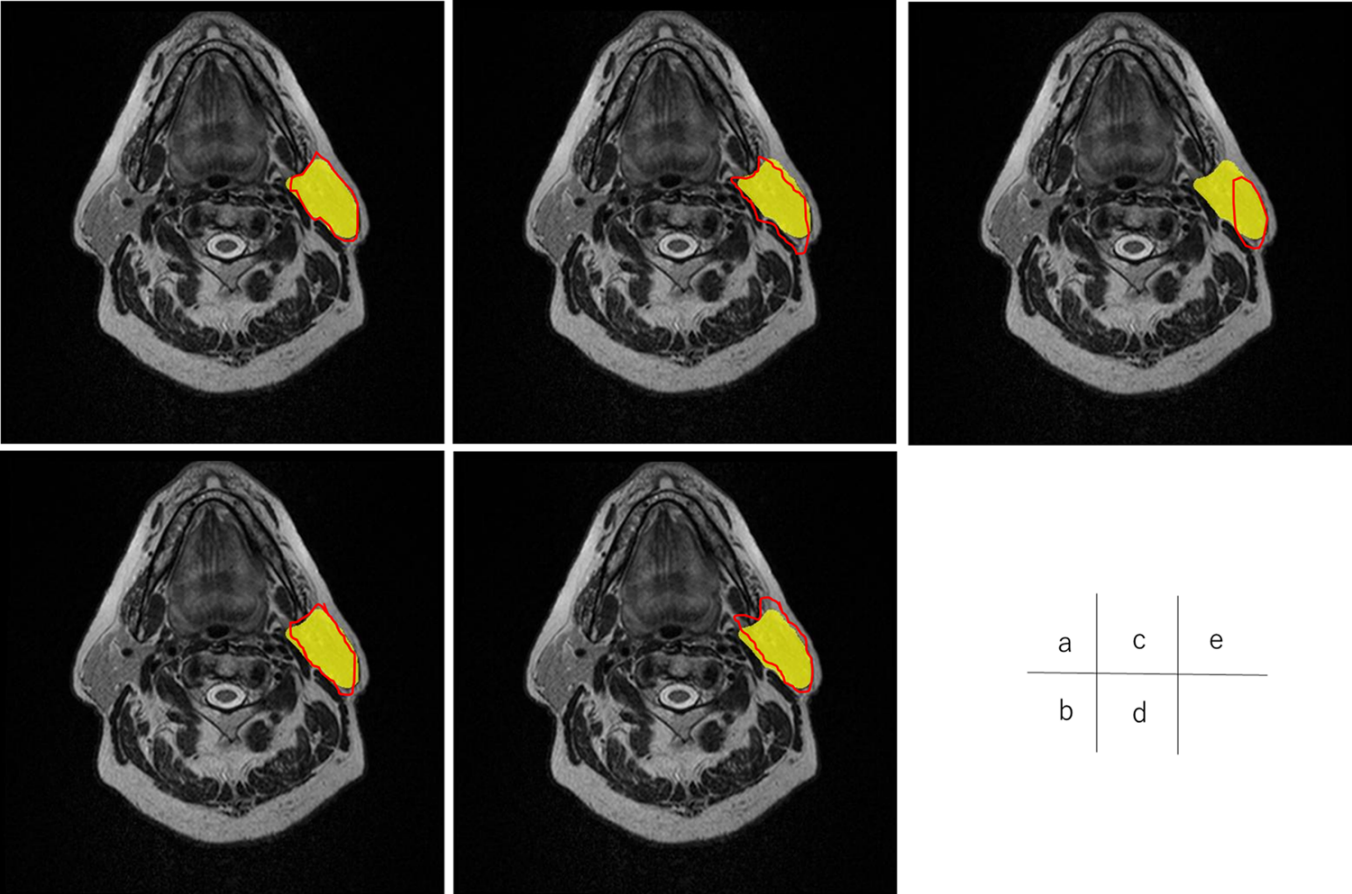
Comparison of manual segmentation and (a) 2.5D generative adversarial network (GAN), (b) 3D GAN, (c) 2.5D U‐net, (d) 3D U‐net, and (e) atlas‐based method in the left parotid glands segmentation. The yellow region shows the reference segmentation, and red line denotes the segmentation by atlas‐based or deep learning methods

**FIGURE 11:**
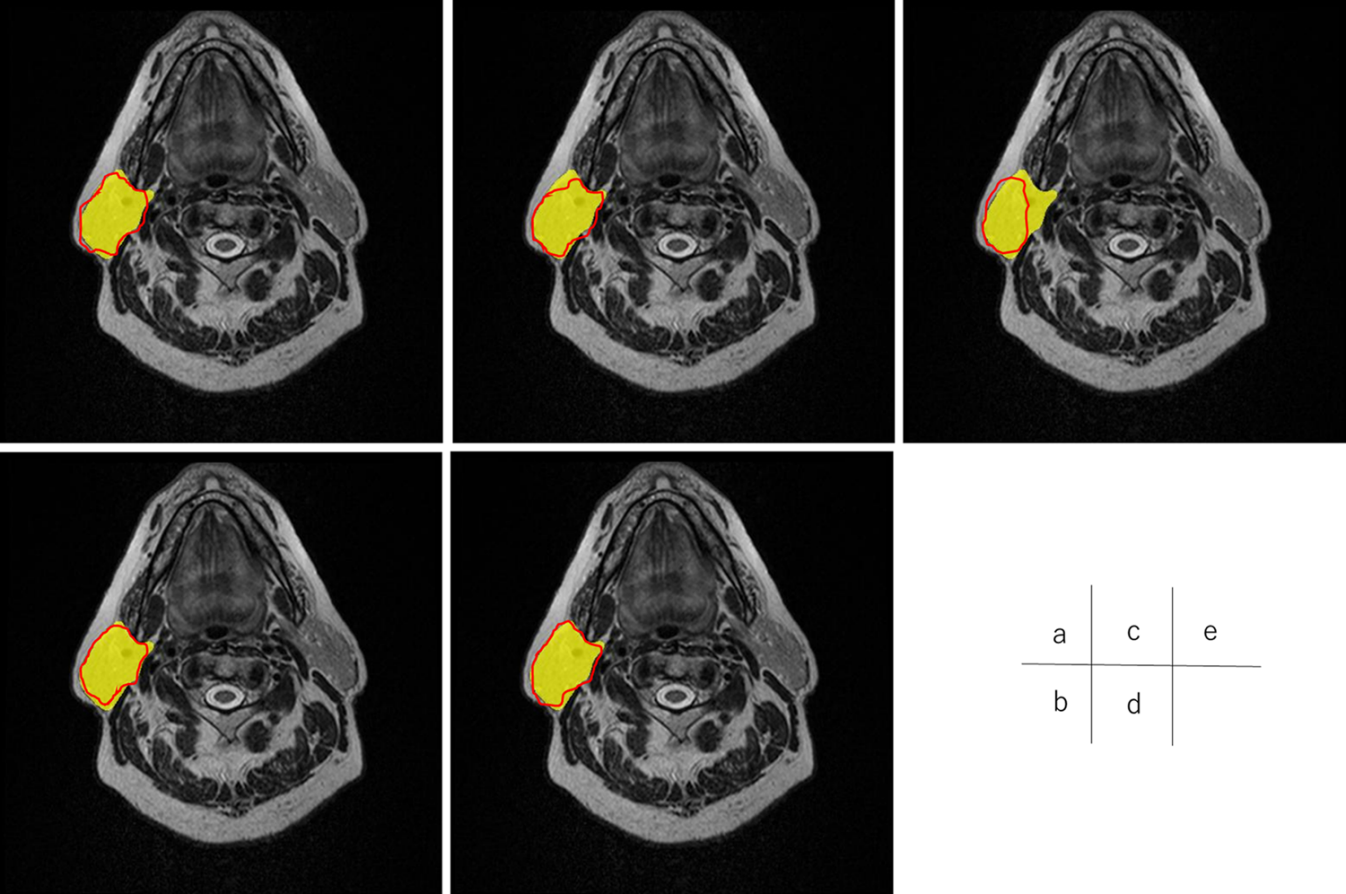
Comparison of manual segmentation and (a) 2.5D generative adversarial network (GAN), (b) 3D GAN, (c) 2.5D U‐net, (d) 3D U‐net, and (e) atlas‐based method in the right parotid glands segmentation. The yellow region denotes the reference segmentation, and red line denotes the segmentation by atlas‐based or deep learning methods

Figures 4, 5, 6, 7, 8, 9, 10, 11 showed the segmentation results of the U‐net model, GAN model, and atlas‐based model for one representative patient. The atlas‐based method underestimated all the segmentations. Comparing the U‐net and GAN models, the 2.5D GAN segmented the bilateral submandibular glands, bilateral lymph node levels II and III, and bilateral parotid glands with high accuracy.

**FIGURE 12:**
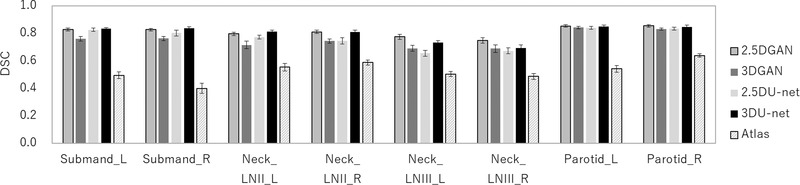
Mean and standard deviation of the Dice similarity coefficient (DSC) values for 2.5D generative adversarial network (GAN), 3D GAN, 2.5D U‐net, 3D U‐net, and atlas‐based methods

Figure 12 shows the results of the DSC, in which manual segmentation is compared with U‐net, GAN, and atlas‐based models. The mean DSC obtained with DL methods of U‐net or GAN was higher than that obtained with the atlas‐based method. There was a significant difference between the DSC values obtained with the DL methods of U‐net or GAN models and that obtained with the atlas‐based method for all OAR segmentations. The DSC of 2.5D GAN was 0.83 for the left submandibular gland, 0.83 for the right submandibular gland, 0.80 for the left lymph node levels II, 0.81 for the right lymph node levels II, 0.77 for the left lymph node levels III, 0.75 for the right lymph node levels III, 0.85 for the left parotid gland, and 0.85 for right parotid gland, which was the highest. The mean DSC with DL methods of U‐net or GAN was higher than that with the atlas‐based method. There was a significant difference between the DSC values of 2.5D GAN and 3D GAN for the bilateral submandibular glands, bilateral lymph node levels II and III, and right parotid gland (*p* < 0.05). A comparison of the 2.5D GAN and 2.5D U‐net revealed that there was a significant difference in the DSC values for the right lymph node levels II and bilateral lymph node levels III (*p* < 0.05). A comparison of the 2.5D GAN and 3D Unet revealed that there was a significant difference in the DSC value for the bilateral lymph node level III (*p* < 0.05). A comparison of 2.5D U‐net and 3D U‐net revealed that the DSC value of the 3D U‐net was significantly higher than that of the 2.5D U‐net for bilateral lymph node level II and bilateral lymph node level III (*p* < 0.05).

**FIGURE 13:**
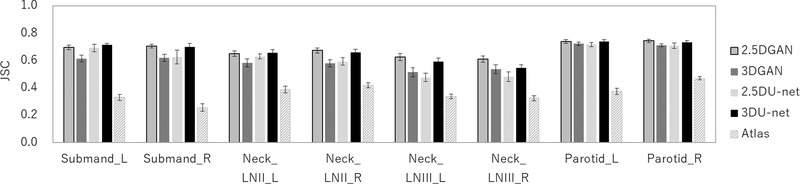
Mean and standard deviation of the Jaccard similarity coefficient (JSC) values for 2.5D generative adversarial network (GAN), 3D GAN, 2.5D U‐net, 3D U‐net, and atlas‐based methods

Figure 13 shows the result of the JSC, which compares the manual segmentation with the U‐net, GAN, and atlas‐based models. The mean JSC with DL methods of U‐net or GAN was higher than that with the atlas‐based method. There was a significant difference between the JSC value with the DL method of U‐net or GAN models and that with atlas‐based method for all OAR segmentations (*p* < 0.05).

The JSC of 2.5D GAN was 0.70 for the left submandibular gland, 0.71 for the right submandibular gland, 0.65 for the left lymph node levels II, 0.68 for the right lymph node levels II, 0.63 for the left lymph node levels III, 0.61 for the right lymph node levels III, 0.74 for the left parotid gland, and 0.75 for the right parotid gland, which was the highest. There was a significant difference between the JSC values of 2.5D GAN and 3D GAN for the bilateral submandibular glands, bilateral lymph node levels II and III, and right parotid gland (*p* < 0.05). A comparison of 2.5D GAN and 2.5D U‐net revealed that there was a significant difference in the JSC values for the right lymph node levels II and bilateral lymph node levels III (*p* < 0.05). A comparison of the 2.5D GAN and 3D U‐net revealed that there was a significant difference in the JSC values for the right lymph node level III (*p* < 0.05). Furthermore, a comparison of 2.5D U‐net and 3D U‐net revealed that the JSC values of 3D U‐net were significantly higher than those of 2.5D U‐net for the right lymph node level II and left lymph node level III (*p* < 0.05).

**FIGURE 14:**
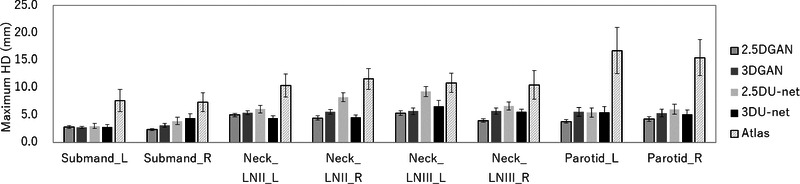
Mean and standard deviation of maximum Hausdorff distance (HD) values for 2.5D generative adversarial network (GAN), 3D GAN, 2.5D U‐net, 3D U‐net, and atlas‐based methods

Figure 14 shows the results of the maximum HD that compares the manual segmentation with U‐net, GAN, and atlas‐based models. The maximum HD with DL methods of U‐net or GAN was lower than that with the atlas‐based method for all OAR segmentations. There was a significant difference between the maximum HD values with the DL method of U‐net or GAN models and that with the atlas‐based method for all OAR segmentations (*p* < 0.05). Furthermore, there was a significant difference between the maximum HD values of 2.5D GAN and 3D GAN for the right submandibular gland, right lymph node level II, right lymph node level III, and left parotid gland (*p* < 0.05). A comparison of 2.5D GAN and 2.5D U‐net revealed that there was a significant difference in the maximum HD of the right submandibular gland, right lymph node level II, and bilateral lymph node level III (*p* < 0.05). Furthermore, a comparison of the 2.5D GAN and 3D U‐net revealed that there was a significant difference in the maximum HD for the right submandibular gland and right lymph node level III (*p* < 0.05). Additionally, a comparison of 2.5D U‐net and 3D U‐net revealed that the maximum HD values of 3D U‐net were significantly higher than those of 2.5D U‐net for the right lymph node level II and bilateral lymph node level III (*p* < 0.05). Table 1 summarized of the comparison of the segmentation performance between 2.5D GAN and the other models of 3D GAN, 2.5D CNN, 3D CNN, and Atlas model.

**TABLE 1:** The comparison of the segmentation performance between 2.5D generative adversarial network (GAN) and the other models of 3D GAN, 2.5D convolutional neural network (CNN), 3D CNN, and atlas model

	2.5D GAN versus 3D GAN	2.5D GAN versus 2.5D CNN	2.5D GAN versus 3D CNN	2.5D GAN versus Atlas
Submand_L	○ (2.5D GAN)	n.s.	n.s.	◎ (2.5D GAN)
Submand_R	◎ (2.5D GAN)	○ (2.5D GAN)	○ (2.5D GAN)	◎ (2.5D GAN)
Neck_LNII_L	○ (2.5D GAN)	n.s.	n.s.	◎ (2.5D GAN)
Neck_LNII_R	◎ (2.5D GAN)	◎ (2.5D GAN)	n.s.	◎ (2.5D GAN)
Neck_LNIII_L	○ (2.5D GAN)	◎ (2.5D GAN)	n.s.	◎ (2.5D GAN)
Neck_LNIII_R	◎ (2.5D GAN)	◎ (2.5D GAN)	◎ (2.5D GAN)	◎ (2.5D GAN)
Parotid_L	○ (2.5D GAN)	n.s.	n.s.	◎ (2.5D GAN)
Parotid_R	◎ (2.5D GAN)	n.s.	n.s.	◎ (2.5D GAN)

*Note*: Submand_L: left submandibular gland; Submand_R: right submandibular gland; Neck_LNII_L: left lymph node levels II; Neck_LNIII_R: right lymph node levels III; Neck_LNIII_R: right lymph node levels III; Parotid_L: left parotid gland; Parotid_R: right parotid gland; ◎: significantly higher DSC, JSC, and smaller HD (*p* < 0.05); 〇, one or two of three metrics had a significantly higher DSC, JSC, and smaller HD (higher performance model).

Abbreviation: n.s., not significant.

## 4 DISCUSSION

The conventional auto‐segmentation tool uses atlas‐based segmentation that builds a library of normal tissue from manual segmentation and extrapolates it to a new patient with rigid or deformable image registration.^34^ Atlas‐based segmentation on the reference image corresponds to the transposition of a new image after a reference image is registered to a new image. The proposed DL methods with U‐net and GAN indicated a more accurate segmentation than the atlas‐based method. It is difficult for the atlas‐based method to correspond to the various body shapes. Conversely, DL, which can adapt to a larger dataset, can aid in improving the statistical power of segmentation. Tong et al. compared the atlas‐based method, model‐based method, and theU‐net for HN segmentation with CT images.^35^ The U‐Net displayed a highly accurate segmentation performance. The current study used only T2‐weighted MRI images. Hague et al. compared the accuracy of auto‐segmentation between MRI and CT images of the HN.^36^ The auto‐segmentation model with MRI images outperformed the model with CT images of the bilateral parotid glands and bilateral submandibular glands. The MRI image exhibited superior visualization of the soft tissue when compared to the CT image, and thus the MRI image was suitable for auto‐segmentation.

Kieselmann et al. compared 2D U‐net, 2.5D U‐net, and 3D U‐net for parotid gland segmentation.^37^ The input image used three adjacent slices for the 2.5D U‐Net. The 2.5D U‐net displayed lower accuracy for the right parotid gland segmentation and higher accuracy for left parotid gland segmentation. The accuracy of the segmentation with the proposed DL methods is equivalent to or slightly higher than that of Kieselmann et al. In the current study, we used 2.5D networks that use patch multi‐slices for an efficient OAR auto‐segmentation method. Kieselmann et al. prepared a patched image that focused on the center of mass of each parotid gland due to limitations in GPU memory. They indicated a limitation that the parotid gland can be omitted in the process of creating the patched image. In this study, a random patch image was created in the slice direction without identifying the geometric position. It plays a role in augmentation. Moreover, 2.5D networks can reduce computation time and consumption of the GPU memory. The accuracy of the segmentation with the 2.5D U‐net did not differ from that of the 3D U‐net for the bilateral parotid glands and bilateral submandibular glands. Conversely, 3D U‐net was superior to 2.5D U‐Net for the segmentation of the lymph node. For segmentation with U‐net, it is necessary to learn the entire 3D shape for the segmentation of the lymph node. Conversely, 2.5D GAN significantly improves the accuracy of the segmentation for most OARs of HN patients when compared to 3D GAN, 2.5D, and 3D U‐nets. Dong et al. proposed U‐Net‐GAN for segmentation of thorax using CT images.^22^ Dong et al. revealed that U‐Net‐GAN improved accuracy of segmentation when compared to U‐Net. Furthermore, Sultana et al. reported that a GAN with a 3D U‐net successfully segmented the pelvic region using CT images.^23^ The 3D network had the advantage that it obtains more spatial information to use entire image volumes. On the other hand, it has the disadvantage that it requires more training patient data to achieve robust performance. In the current study, the 3D U‐net had better segmentation performance than 2.5D U‐net. Thus, the effect of the number of sample size between 3D U‐net and 2.5D U‐net were small and the difference of the spatial information for the training may be dominant. On the other hand, the 2.5D GAN showed better segmentation performance than 3D GAN. The generator used U‐net which provides the image requires more spatial information. The GAN uses the discriminator in addition to the generator. The discriminator that distinguishes the ground truth and the segmentation created by the generator would be required fine training with the augmentation of the patched images. The proposed 2.5D GAN contributes to accurate segmentation via providing trained parameters and fine distinguishing between real and fake segmentations.

A previous study reported that there was a significant difference in mean volumes between five HN cancer expert oncologists despite the use of accepted delineation guidelines.^38^ The current study evaluated the interobserver variability of the segmentation. The minimum DSC values between the manual segmentation data included in RT‐MAC 2019 dataset and manual segmentation by radiation observers were 0.80. Therefore, the accuracy of the manual segmentation and interobserver variability can be dominant to the uncertainty of segmentation with DL. However, auto‐segmentation aids in decreasing the interobserver variability, the time, and cost of treatment planning by using reference segmentation with sufficient levels.

The proposed model may be useful for MRI‐based planning. GAN can perform image synthesis such as CT‐to‐MRI.^39^ In further studies, we will use the proposed model that synthesized MRI images from the CT images to enhance accuracy of the segmentation with the CT image. The European SocieTy for Radiation and Oncology ‐Advisory Committee on Radiation Oncology Practice (ESTRO‐ACROP) reported the limitations and benefits of MRgRT.^40^ They recommended developing data‐intensive computer‐based solutions, such as auto‐segmentation with DL, and supporting medical decisions with radiomics analysis. The proposed model can aid in the online adaptive workflow of the MRgRT. The limitation of the current study corresponds to the evaluation of the dosimetric effect via auto‐segmentation. With respect to clinical implementation, an evaluation of dosimetric errors with manual and auto‐segmentation will be performed in future studies. Moreover, the current study has difficulty comparing their result with the competition participants because the RT‐MAC challenge already finished. Additionally, the available structures were limited. The current study showed a 2.5D GAN that used patched images has a possibility to improve the accuracy of the segmentation than conventional atlas‐based, U‐net, and 3D GAN segmentations. Further study will be performed to improve the applicable 2.5D GAN model for the other structures such as the brainstem, chiasm, optic nerves, and larynx.

## 5 CONCLUSION

In the current study, we investigated auto‐segmentation of the OAR for HN patients with U‐net and GAN models on MR images. The results indicated that the 2.5D GAN‐based segmentation is superior to conventional U‐net‐based and atlas‐based segmentation. Our proposed model is potentially valuable in terms of improving the efficiency of HN radiotherapy treatment planning.

## CONFLICT OF INTEREST

Masato Tsuneda's institution: MR Linac ART division is an endowment department, funded by Elekta. Other authors have no conflict of interest to disclose.

## FUNDING INFORMATION

National Cancer Center Research and Development, Grant Number: 2020‐J‐3

## ETHICS STATEMENT

All procedures performed in studies involving human participants were in accordance with the ethical standards of the institutional and/or national research committee and with the 1964 Helsinki declaration and its later amendments or comparable ethical standards.

## AUTHOR CONTRIBUTION

Daisuke Kawahara and Masato Tsuneda involved in designing this study. The deep learning network was designed by Daisuke Kawahara. The manuscript was drafted by Daisuke Kawahara, Masato Tsuneda, Hiroyuki Okamoto, Mitsuhiro Nakamura, Teiji Nishio, and Yasushi Nagata.

## Data Availability

The data that support the findings of this study can be obtained at https://doi.org/10.7937/tcia.2019.bcfjqfqb.^41^
